# Phase I study of [^131^I] ICF01012, a targeted radionuclide therapy, in metastatic melanoma: MELRIV-1 protocol

**DOI:** 10.1186/s12885-022-09495-3

**Published:** 2022-04-15

**Authors:** Emilie Thivat, Jacques Rouanet, Philippe Auzeloux, Nicolas Sas, Elodie Jouberton, Sophie Levesque, Tommy Billoux, Sandrine Mansard, Ioana Molnar, Marion Chanchou, Giovanna Fois, Lydia Maigne, Jean-Michel Chezal, Elisabeth Miot-Noirault, Michel D’Incan, Xavier Durando, Florent Cachin

**Affiliations:** 1 INSERM U1240 IMoST, Université Clermont Auvergne, F-63000 Clermont-Ferrand, France; 2 Département de Recherche Clinique, Délégation Recherche Clinique et Innovation, Centre Jean PERRIN, F-63000 Clermont-Ferrand, France; 3 Centre d’Investigation Clinique UMR501, F-63000 Clermont-Ferrand, France; 4 Service de Dermatologie et d’Oncologie Cutanée, CHU Clermont-Ferrand, F-63000 Clermont-Ferrand, France; 5 Service de Physique Médicale, Centre Jean PERRIN, F-63000 Clermont-Ferrand, France; 6 Service de Médecine Nucléaire, Centre Jean PERRIN, F-63000 Clermont-Ferrand, France; 7 Unité de radiopharmacie, Centre Jean PERRIN, F-63000 Clermont-Ferrand, France; 8 Laboratoire de Physique de Clermont UMR6533, CNRS/IN2P3, Université Clermont Auvergne, Clermont-Ferrand, France; 9 Département d’oncologie médicale, Centre Jean PERRIN, F-63000 Clermont-Ferrand, France

**Keywords:** [^131^I]ICF01012, Targeted radionuclide therapy, Metastatic melanoma, Dosimetry

## Abstract

**Background:**

Benzamide-based radioligands targeting melanin were first developed for imaging melanoma and then for therapeutic purpose with targeted radionuclide therapy (TRT).

[^131^I]ICF01012 presents a highly favorable pharmacokinetics profile in vivo for therapy. Tumour growth reduction and increase survival have been established in preclinical models of melanoma. According the these preclinical results, we initiate a first-in-human study aimed to determine the recommended dose of [^131^I]ICF01012 to administer for the treatment of patients with pigmented metastatic melanoma.

**Methods:**

The MELRIV-1 trial is an open-label, multicentric, dose-escalation phase I trial. The study is divided in 2 steps, a selection part with an IV injection of low activity of [^131^I]ICF01012 (185 MBq at D0) to select patients who might benefit from [^131^I]ICF01012 TRT in therapeutic part, i.e. patient presenting at least one tumour lesion with [^131^I]ICF01012 uptake and an acceptable personalized dosimetry to critical organs (liver, kidney, lung and retina). According to dose escalation scheme driven by a Continual Reassessment Method (CRM) design, a single therapeutic injection of 800 MBq/m^2^, or 1600 MBq/m^2^, or 2700 MBq/m^2^ or 4000 MBq/m^2^ of [^131^I]ICF01012 will be administered at D11 (± 4 days). The primary endpoint is the recommended therapeutic dose of [^131^I]ICF01012, with DLT defined as any grade 3-4 NCI-CT toxicity during the 6 weeks following therapeutic dose. Safety, pharmacokinetic, biodistribution (using planar whole body and SPECT-CT acquisitions), sensitivity / specificity of [^131^I]ICF01012, and therapeutic efficacy will be assessed as secondary objectives. Patients who received therapeutic injection will be followed until 3 months after TRT. Since 6 to 18 patients are needed for the therapeutic part, up to 36 patients will be enrolled in the selection part.

**Discussion:**

This study is a first-in-human trial evaluating the [^131^I]ICF01012 TRT in metastatic malignant melanomas with a diagnostic dose of the [^131^I]ICF01012 to select the patients who may benefit from a therapeutic dose of [^131^I]ICF01012, with at least one tumor lesion with [^131^I]ICF01012 uptake and an acceptable AD to healthy organ.

**Trial registration:**

Clinicaltrials.gov: NCT03784625. Registered on December 24, 2018.

Identifier in French National Agency for the Safety of Medicines and Health Products (ANSM): N°EudraCT 2016-002444-17.

## Background

Melanoma is a cancer arising from skin melanocytes but mucosal or uveal forms exist [1]. Surgical excision represents the first-line treatment for melanoma [2]. Depending on TNM staging, adjuvant immunotherapy or targeted therapy could be proposed to lower the metastatic risk [3]. However, melanoma is a highly metastatic cancer and represents a major public health problem for many years. Until 2010, only chemotherapies were available to treat metastatic melanomas and prognostic was poor [4]. MAPK pathway targeted therapies including BRAF V600E [5] and MEK-activated proteins [6] and immunotherapies with antibody against immune check-point inhibitors such as CTLA-4 [4], PD-1 [7] and its ligand PD-L1, have completely modified metastatic melanoma prognosis. However, patients can be non-responders or develop some resistance [8]. Therefore, it is still mandatory to develop new therapeutic strategies as external stereotactic radiation therapy, alone or combined with systemic treatments. Targeted radionuclide therapy (TRT) [9], a nuclear medicine strategy that consists in administering a radiopharmaceutical compound to the patient in order to deliver a high absorbed dose of radiation to tumours while avoiding or minimizing normal tissue toxicities, could be one of them.

Multiples approaches have been developed in this field [9, 10], using melanin targeting by antibodies or small molecules (benzamides), or MSH analogs (peptides), or membrane receptors targeting (antibodies). A few have been tested in human with mixed results [9, 10]. Only a phase I trial has tested a benzamide radiolabeled with iodine-131 on a series of 26 patients with metastatic malignant melanoma [11]. All received a diagnostic dose of 235 ± 62 MBq of [^123^I]-BA52 for planar and SPECT/CT imaging, and only 9 patients were selected to receive a radionuclide therapy with a single dose of [^131^] I-BA52 with different activity levels between 510 to 6600 MBq. A specific uptake and long-term retention in tumor tissue with low transient uptake in the excretory organs was observed with diagnostic injection of [^123^] I-BA52. The highest estimated dose to a normal organ was found for the lung (mean, 3.1 Gy/GBq). No relevant acute or mid-term toxicity was observed with the administered doses. In tumor tissue, a maximum dose of 12.2 Gy per GBq of [^131^] I-BA52 was calculated. Among the 5 patients who received [^131^] I-BA52 dose > 4.3 GBq (potentially effective), 3 demonstrated a surprisingly long survival of more than 2 years. These results highlight the potential of small molecule with high melanin affinity for TRT to treated metastatic melanoma.

Since 1990’s, our team has developed several compounds displaying a high affinity for melanin, benzamide-derived melanin-targeting molecules for imaging and/or therapy [12–14]. BZA and BZA2 radiolabeled with iodine-123 have been clinically tested for a diagnostic approach for SPECT imaging of metastatic melanoma leading to phase II clinical trials [15, 16]. In a prospective and multicentric phase III clinical study comparing [^123^I]BZA2 scintigraphy to [^18^F]-FDG PET-CT for melanoma staging (*n* = 87), the sensitivity and specificity of BZA2 for the diagnosis of pigmented lesions were 85 and 76% [17].

ICF01012 is a pharmacomodulated form of this BZA2, with highly favorable pharmacokinetics in murine models for therapy, with a long-lasting tumoral intake (8 days) and a rapid elimination of non-target organs (< 24 h) [18]. The TRT efficiency of [^131^I]ICF01012 have been achieved on syngeneic murine models (B16F0 and B16Bl6) and murine model bearing xenografts (SK-Mel 3). With single or multiple injection of [^131^I]ICF01012, a slowing of tumor growth and an increase in survival were observed [19–21]. A clear relationship between the presence of melanins and the efficacy of therapy has also been demonstrated. Radiotoxicity studies conducted with [^131^I]ICF01012 at a therapeutic dose on highly pigmented mouse models have shown a transient decrease in white blood cells and a relative decrease in the thickness of the retina [22]. Dosimetry extrapolations have shown retina-values below the maximum tolerated doses, suggesting that this ocular specific targeting is not expected to have an impact in human [23]. Furthermore, preliminary toxicological studies point out that TRT with [^131^I]ICF01012 was compatible with clinical requirements. Impact of TRT using [^131^I]ICF01012 has also been evaluated on metastasis mechanisms [24] and current melanoma treatments (immune checkpoints inhibitors [25] and MAPK targeted therapies [26]) in murine melanoma models.

According to these preclinical results, [^131^I]ICF01012 could be a promising theranostic approach with a first diagnostic dose of [^131^I]ICF01012 (low activity) for pre-selected melanin positive melanoma patients who might benefit from this [^131^I]ICF01012 TRT (therapeutic activity). The MELRIV-1 trial is a first-in-human study aimed to determine the recommended dose of [^131^I]ICF01012 to administer for the treatment of patients with pigmented metastatic melanoma (binding [^131^I]ICF01012).

## Methods/design

### Study design

This is an open-label, multicentric, “first-in-human”, dose-escalation phase I trial of a radiopharmaceutical.

The study based on a theranostic approach is divided in 2 steps:

- Firstly, a “selection part”, to determine if the patient is eligible for therapy, i.e., if the radiopharmaceutical is up-taken by metastases. During this phase, a single IV injection of low activity of [^131^I]ICF01012, called “diagnostic dose” will be injected to the patient. Pre-treatment dosimetry allows to measure for each patient the absorbed dose by organs and tissues. Patients will be eligible for the second step, the “therapeutic part” if they present at least one tumor lesion with [^131^I]ICF01012 uptake and if mean absorbed dose (AD) to liver < 26 Gy, kidney AD < 16.2 Gy, lung AD < 15 Gy et retina AD < 40 Gy.
- Secondly, a “therapeutic part” consisting of a single IV injection of [^131^I]ICF01012 at a therapeutic activity. The dose escalation will be driven by a Continual Reassessment Method (CRM) [27] with 4 [^131^I]ICF01012 activity levels: 800, 1600, 2700, or 4000 MBq/m^2^.

The duration of intervention for each patient will be 7 days (diagnostic phase only) or 3 weeks (diagnostic phase + therapeutic phase). Follow-up will last 4 weeks (diagnostic phase only) or 10 weeks (diagnostic phase + therapeutic phase).

### Coordination and participating institutions

The Centre Jean Perrin is the sponsor and responsible for the coordination of the trial, data management, monitoring and statistical analysis.

The multicenter study is currently conducted at 2 sites in France: the University Hospital of Clermont-Ferrand and the Centre Jean Perrin. Patients with cutaneous melanomas will be recruited at the Dermatology and Oncodermatology Department of the University Hospital of Clermont-Ferrand and patients with uveal melanomas will be recruited at the Jean Perrin Cancer Center. For all patients, targeted radionuclide therapy will be performed at the Nuclear Medicine Department of the Jean Perrin Cancer.

### Objectives and endpoints

#### Main objective

The primary objective is to determine the recommended dose of [^131^I]ICF01012 to use for pigmented melanoma metastases TRT.

The primary endpoint is the recommended therapeutic dose of [^131^I]ICF01012, defined as the higher dose for which the percentage of dose-limiting toxicity (DLT) is lower than 33%. DLT is defined as any grade 3-4 NCI-CT toxicity, except for alopecia, nausea and vomiting or fever, which can be managed by symptomatic treatment. Only toxicities experienced during the 6 weeks following therapeutic dose administration will be considered as DLT.

#### Secondary objectives

The secondary objectives and the corresponding end-points are:

- To determine the [^131^I]ICF01012 sensitivity and specificity in a patient-based analysis and in a lesion-based analysis.
  [^131^I]ICF01012 sensitivity and specificity in patient-based analysis and in lesion-based analysis will be quantified by comparison of visualized or non-visualized lesions with [^131^I]ICF01012 imaging compared to metastatic lesions objectified by standard imagery (TEP-TDM or TDM) and or clinical evaluation.
- To evaluate the tolerance of [^131^I]ICF01012. [^131^I]ICF01012 tolerance (acute and delayed toxicities) will be evaluated with the Common Terminology Criteria for Adverse Events (CTCAE, version 4.03) with a special consideration for skin-related, ocular and neurological toxicities. Tolerance assessments include: Clinical examination with vitals (heart rate, blood pressure, temperature, respiratory rate), weight, height, OMS performance status; Cardiac evaluation (myocardial perfusion scintigraphy with measure of left ventricular ejection fraction (LVEF), Electrocardiogram (ECG); Specialised consultation with dermatologist, neurologist and ophthalmologist (with of a dilated fundus examination); Blood test including Fool blood count, prothrombine time, factor V, sodium, potassium, chloride, calcium, phosphore, bicarbonate, serum total protein, serum albumin, fasting blood glucose level, Thyroid-stimulating hormone (TSH), free thyoroxine (fT4), free triiodothyronine (fT3), creatinine, urea, bilirubin, Alkaline phosphatase (ALP), Gamma-glutamyltransferase (GGT), Aspartate amino transferase (AST or SGOT), Alanine amino transferase (ALT or SGPT), Troponin Lactate dehydrogenase (LDH); 24-h urine collection tests: glucose, urea, creatinine, albumin, sodium, potassium, magnesium, calcium, protein electrophoresis. Reporting of serious adverse events and suspected unexpected serious adverse reaction will be carried out according to the local regulations.
- To evaluate the pharmacokinetics of [^131^I]ICF01012 including the study of biodistribution and excretion.
  Variations of [^131^I]ICF01012 radioactivity concentration (AUC, Tmax and Cmax) will be measured on whole blood plasma during 8 days after diagnostic dose. Plasmatic free fraction of [^131^I]ICF01012 will be quantified by radioactive counting for each patient.
  24-h urinary excretion will be measured by counting.
- The biodistribution will be assessed with planar scintigraphy by measuring the percentage of [^131^I]ICF01012 injected dose absorbed by target and non-target organs for both diagnostic and therapeutic doses.
- To assess the therapeutic response of [^131^I]ICF01012. Therapeutic response (objective response rate, rate of clinical benefit, progression-free survival) will be assessed by TDM according to RECIST 1.1 criteria and by [^18^F] FDG TEP/TDM according to PERCIST criteria.
- To evaluate the correlation between therapeutic response, injected dose and tumoral uptake.
- To evaluate the personalized dosimetry applied to [^131^I]ICF01012, and its interindividual variability, Absorbed dose for each organ will be assessed by MIRD formalism.
- To compare the therapeutic recommended dose with the dose calculated with personalized dosimetry;
- To compare biodistribution of diagnostic dose and therapeutic dose.

### Patients

#### Inclusion criteria

- Patient with metastatic melanoma failure to recommended treatments by French Health Authority
- Initial histological diagnosis of cutaneous melanoma pigmented or unknown status or choroidal melanoma
- Presence of at least one measurable lesion and / or evaluable in 18FDG-PET as PERCIST criteria
- Presence of at least one measurable lesion and / or evaluable as CT RECIST 1.1 criteria
- WHO performance index ≤2
- Age > 18 years
- Life expectancy > 3 months

#### Exclusion criteria

- Symptomatic brain metastases
- Patient with a VI skin phototype
- Previous treatment with chemotherapy, radiotherapy, immunotherapy and targeted therapy in the previous 4 weeks, the first injection of [^131^I]ICF01012
- Pregnant woman, nursing or woman of childbearing age refusing to follow effective contraception during treatment and 12 months after the administration of therapeutic dose
- Man refusing to follow effective contraception during treatment and 12 months after the administration of therapeutic dose
- Other evolutionary known cancer in the past 5 years
- Earlier irradiation of more than 25% of the bone marrow
- Suspicion of invasion of more than 25% of the bone marrow on imaging 18F-FDG PET-CT
- External Radiotherapy on target organs or the maximum dose as recommended in force
- Uncontrolled diabetes
- Known history of allergy to the excipients of the solution of [^131^I]ICF01012
- Any comorbidity or severe disease at the discretion of the investigator

### Intervention

#### Study drug

[^131^I]N-(2-diethylaminoethyl)-6-iodoquinoxaline-2-carboxamide ([^131^I]ICF01012) is a quinoxaline derivative radiolabeled with iodine-131. [^131^I]ICF01012 is synthesized by radio-iododestannylation in presence of hydrogen peroxide as oxidative agent. Production takes place in the radiopharmacy unit of the Jean Perrin Center. For radiation protection, the synthesis is carried out entirely on an AIO® synthesizer (TRASIS).

The experimental drug is an injectable solution of [^131^I]ICF01012 (mixture of 0.9% NaCl saline solution and ethanol / water). The maximum injectable volume is 10 mL. The radiochemical purity of radiolabeled [^131^I]ICF01012 is required to be over 95% before injection.

#### Study drug and premedication administration

##### Selection part

The diagnostic dose of [^131^I]ICF01012 labeled with an activity of 185 MBq will be administered by slow intravenous injection in a approximatively 10 min at D0 in the presence of a nuclear medicine physician.

Patients will receive per os premedication of potassium iodide 130 mg of 48 h before [^131^I]ICF01012 injection, and of loratadine 10 mg and potassium iodide 130 mg, 24 h before [^131^I]ICF01012 injection. The day of the injection, oral potassium iodide 130 mg will be taken approximatively 4 h before injection and IV premedication (dexchlorpheniramine 5 mg and hydrocortisone hémisuccinate 500 mg) 5 minutes before injection. Thyroid blockade will be pursued for a period of 8 days (D1-D8) (potassium iodide 130 mg daily).

##### Therapeutic part

According to the dose escalation scheme, a single therapeutic injection of 800 MBq/m^2^, or 1600 MBq/m^2^, or 2700 MBq/m^2^ or 4000 MBq/m^2^ of [^131^I]ICF01012 will be administered at D11 (± 4 days) under the same conditions as those for diagnostic injection (slow IV injection in approximatively 10 min).

Before therapeutic injection of [^131^I]ICF01012, patients will receive identical premedication as for the diagnostic injection: potassium iodide 130 mg at 48 h before infusion, loratadine 10 mg and potassium iodide 130 mg 24 h before injection. The day of the injection, oral potassium iodide 130 mg will be taken approximatively 4 h before injection and IV premedication (dexchlorpheniramine 5 mg and hydrocortisone hémisuccinate 500 mg) 5 minutes before injection. Thyroid blockade will be pursued for a period of 60 days (D12-D71) (potassium iodide 130 mg daily).

#### Study procedures and participant timeline

The overview of study assessments and procedures is detailed in Table 1 for patients who participate only to the selection part, and in Table 2 for patients who participate to the selection part and the treatment part.

**Table 1:** Assessment schedule for patients who received diagnostic injection and were not eligible to therapeutic part

TIMEPOINT	Screenig / baseline	Selection part			Follow-up period		
Week		W0			W1	W2	W3
Day	−30 to −1	D0	D1	D4	D7	D14 ± 4	D35 ± 4
Informed consent	✓						
Medical history and demographic	✓						
Prior / concomitant medication	✓	Collecting during all the study					
Adverse event evaluation	✓	Collecting during all the study					
Physical exam	✓	✓	✓	✓	✓	✓	✓
Vital signs	✓	✓before, 5 min, 30 min, 1 h, 3 h after	✓	✓	✓	✓	✓
Performance status OMS	✓	✓			✓	✓	✓
ECG	✓						✓
LVEF	✓						
Dermatologist consultation	✓					✓	
Ophthalmologist consultation	✓					✓	
Neurologist consultation	✓					✓	
Imaging acquisition for dosimetry		✓30 min, 1 h, 3 h after	✓	✓	✓		
Disease evaluation	✓						
Pregnancy test	✓						
Hematology and coagulation	✓			✓	✓	✓	✓
Blood chemistry	✓			✓	✓	✓	✓
Thyroid function	✓						✓
Urinalysis	✓					✓	✓
Pharmacokinetic sample		✓Before, 5, 10, 15, 30 min, 1 h, 3 h after	✓	✓	✓		
[131I]ICF01012		✓ 185 MBq

**Table 2:** Assessment schedule for patients who received diagnostic and therapeutic injection

TIMEPOINT	Screenig / baseline	Selection part				Therapeutic part						Follow-up period				
Week		W0				W1					W2	W3	W5	W7	W10	W13
Day	−30 to −1	D0	D1	D4	D7	D11 ± 4	D12 ± 4	D13 ± 4	D15 ± 4	D16 ± 4	D18 ± 4	D25 ± 4	D39 ± 4	D53 ± 4	D74 ± 4	D96 ± 4
Informed consent	✓															
Medical history and demographic	✓															
Prior / concomitant medication	✓	Collecting during all the study														
Adverse event evaluation	✓	Collecting during all the study														
Physical exam	✓	✓	✓	✓	✓	✓	✓	✓	✓	✓	✓	✓		✓	✓	✓
Vital signs	✓	✓ before, 5, 30 min, 1 h, 3 h after injection	✓	✓	✓	✓ before, 5, 30 min, 1 h, 3 h after injection	✓	✓	✓	✓	✓	✓		✓		✓
Performance status OMS	✓	✓			✓	✓	✓	✓	✓	✓	✓	✓		✓		✓
ECG	✓													✓		✓
LVEF	✓															✓
Dermatologist consultation	✓											✓		✓		✓
Ophthalmologist consultation	✓				OCT							✓OCT				✓
Neurologist consultation	✓											✓				
Imaging acquisition for dosimetry		✓30 min, 1 h, 3 h after	✓	✓	✓				✓		✓	✓			✓	
Disease evaluation	✓															✓
Pregnancy test	✓															
Hematology and coagulation	✓			✓	✓				✓	✓	✓	✓	✓	✓		✓
Blood chemistry	✓			✓	✓		✓		✓	✓	✓	✓	✓	✓		✓
Thyroid function	✓					✓							✓	✓		✓
Urinalysis	✓					✓	✓		✓	✓	✓	✓	✓	✓		✓
Pharmacokinetic sample		✓Before, 5, 10, 15, 30 min, 1 h, 3 h after														
[131I]ICF01012		✓ 185 MBq				✓ TI^a^										

^a^a single therapeutic injection (TI) of 800 MBq/m^2^, or 1600 MBq/m^2^, or 2700 MBq/m^2^ or 4000 MBq/m^2^ of [131I]ICF01012

Before any study-related assessment starts, written informed consent will be obtained for each patient. A screening/baseline visit will include imaging for disease evaluation and a dosimetric whole-body CT-scan within 30 days before administration of the diagnostic dose (D0). Within 15 days before administration of the diagnostic dose, the patients will have a clinical evaluation with vital signs, cardiac evaluation, specialized consultation with a dermatologist, a neurologist and an ophthalmologist, blood and urine test and a pregnancy test (for women of childbearing age).

Patients are registered before the diagnostic dose (D0).

##### Selection part

For the selection part, patients will be able to stay hospitalized for 24 h after injection at their request. The diagnostic dose will be injected at D0 with a follow-up of vital signs: before, 5 min after the start of infusion, at the end of infusion, 30 min, 1 h, 3 h, and 24 h (D1) after end of infusion and then at D4 and D7.

The in vivo biodistribution of [^131^I]ICF01012 will be assessed using whole-body planar scintigraphy acquisition (30 min, 1 h, 3 h at D0, 24 h (at D1) 4 days (D4) and 7 days (D7) after injection) and Single Photon Emission Computed Tomography/Computed Tomography (SPECT-CT) acquisitions at 3 h (D0), 24 h (D1), 4 days after injection (D4) and SPECT at D7.

Blood samples for pharmacokinetic study will be collected at 5, 10, 15, 30 min, 1 h, 3 h (D0) 24 h (D1), 96 h (D4) and 168 h (D7) post-injection. A 24-h urine collection will be realized for hospitalized patients for excretion evaluation. Blood and urine tests for safety evaluation will be performed at D4 and D7.

The eligibility criteria for therapeutic part (at least one metastasis with [^131^I]ICF01012 uptake and an acceptable AD to major healthy organs) will be checked from D4 until D7 with dosimetry results. After verification of the eligibity criteria for therapeutic part, a dose allocation request will be done using an eCRF. Eligible patients for therapeutic phase will have an ocular optical coherence tomography (OCT) before therapeutic injection.

##### Therapeutic part

A unique therapeutic injection of [^131^I]ICF01012 at a dose level of 800 MBq/m^2^, or 1600 MBq/m^2^, or 2700 MBq/m^2^ or 4000 MBq/m^2^ (according to dose escalation scheme), will be administered D11 ± 4 days with a follow-up of vital signs: before, 5 min after the start of infusion, at the end of infusion, 30 min, 1 h, 3 h, and 24 h (D12) after the end of infusion and then 2 days, 4 days, 5 days and 7 days after therapeutic dose. Patients will have to stay hospitalized between 3 and 7 days after injection, depending on received radioactivity, Patients will be discharged when 1-m rate of exposure reaches <40μSievert.h^− 1^.

The biodistribution of [^131^I]ICF01012 will be assessed using Whole-body planar scintigraphy acquisition (4 days (D15 ± 4 days), 7 days (D18 ± 4 days), 14 days (D25 ± 4 days), 63 days (D74 ± 4 days) after therapeutic injection) and SPECT-CT acquisition 4 days after injection (D15 ± 4 days) and SPECT at D77 days (D18 ± 4 days) 14 days (D25 ± 4 days), 63 days (D74 ± 4 days) after therapeutic injection. Blood and urine tests for safety evaluation will be performed 1 day, 4 days, 5 days and 7 days after therapeutic dose.

##### Follow-up

Non-eligible patients for therapeutic part will be followed during 3 weeks: 2 follow-up visits are scheduled 14 days and 35 days after diagnostic dose injection for safety evaluation (clinical examination, vital measurement, blood and urinary analyses, specialized consultations (ophthalmologist, neurologist and dermatologist)).

For patient receiving the therapeutic dose, five follow-up visits are scheduled 14, 28, 42, 63 and 85 days after therapeutic dose injection. Each visit will include: clinical examination, vital measurement, blood and urinary analyses. Specialized consultations (ophthalmologist, neurologist and dermatologist) will occur 14 days after therapeutic dose and for dermatologist consultations 42 and 85 after therapeutic dose. Dosimetric acquisitions will be done on 14 and 63 after therapeutic dose (see below). Post-therapeutic cardiac evaluation (myocardial perfusion scintigraphy) and disease staging (cranial and thoraco-abdominopelvic CT scan and [^18^F] FDG PET-CT) will be done 85 days after therapeutic dose.

### Personalized dosimetry

The in vivo biodistribution of [^131^I]ICF01012 will be assessed using planar whole body (2D) and thorax-abdomen-pelvis SPECT-CT acquisitions (3D). Planar and SPECT acquisitions will be performed following the Medical Internal Radiation Dose Committee (MIRD) guidelines for quantitative imaging with ^131^I [28, 29] on a Symbia Intevo T6 SPECT/CT (Siemens), equipped with HEGP collimators and 3/8″ NaI detector crystals. The time-integrated activity $\overset{\sim }{A}\left({r}_S\right)$ (or total number of nuclear transformations in source region *r*_*S*_) will be calculated by fitting time-activity curves for each region previously segmented on CT scan. The time-integrated activity obtained for diagnostic part is then scaled with the expected injected activity for therapeutic part. The patient specific S-values (defined as absorbed dose in target region *r*_*T*_ per nuclear transformation in source region *r*_*S*_) will be computed by Monte-Carlo simulation using GATE. The mean absorbed dose to organs and tumours will be calculated using MIRD formalism [21]. According to this formalism, the mean absorbed dose is given by:

$$D\left({r}_T\right)=\sum_{r_S}\overset{\sim }{A}\left({r}_S\right)S\left({r}_T\leftarrow {r}_S\right)$$

The most unfavourable result of the dosimetry based on 2D or 3D imaging quantification will be retained.

### Statistical considerations

#### Sample size

Using a CRM model with 4 dose levels, i.e. with cohorts of one patient by dose level and at least 6 patients at the recommended dose, a minimum of 6 patients and a maximum of 18 patients should be included in the “therapeutic part”. Sample size calculation for the selection part is based on the hypothesis that 50% of patients will have at least an [^131^I]ICF01012 lesion uptake as observed with BZA2 in phase III study [8] and thus be eligible for the therapeutic part. Therefore, between 12 to 36 patients will be enrolled in the selection part in order to ensure the necessary sample size for the therapeutic part (between 6 to 18).

#### Data analysis

Due to the nature and the design of the study, statistical analysis will be mainly descriptive. Two populations will be analyzed: the diagnostic population (all participants who receive the diagnostic dose) and the therapeutic population (all participants who receive the therapeutic dose). The recommended therapeutic dose will be defined as the highest tested dose for which the percentage of DLT in the diagnostic population is lower than 33%. DLT percentage will be calculated for each tested dose. Only therapeutic population will be included in the analysis of DLT and the efficacy analysis. The statistical significance threshold is set at 5%. Statistical analyses will be performed using R software.

### Data management and monitoring

Study data will be entered by investigator or delegate in an eCRF based on the Web-Based Data Capture (WBDC) system “Ennov Clincal”. Health-related personal data captured during this study are strictly confidential and accessible only by investigators and authorized professionals who are subject to professional secrecy. The investigator ensures that pseudonymized patient data are recorded on eCRF and of the provision of answers to data queries.

Monitoring reviews (site monitoring and central data monitoring) will be regularly carried out by a clinical research associate mandated by the sponsor to ensure the respect of the study protocol and procedures therein, and the quality of the collected data (accuracy, missing data, consistency with the source documents). Monitoring reports will ensure traceability.

### Independent data monitoring Committee (IDMC)

IDMC will be set up to assess the progress of the clinical trial, safety data, benefit/risk ratio of the trial and to recommend to the Sponsor whether to continue, modify, or stop the trial. Members composing the IDMC will be selected to have the relevant clinical trials/medical expertise without direct involvement in the conduct of the trial. The committee will include a nuclear physician and 2 oncologists. Given the very low number of expected DLT and the use of a CRM, the committee will meet for each observed DLT or every 5 patients treated at therapeutic dose in absence of DLT.

### Trial status

The MELRIV-1 trial is currently recruiting. Participant recruitment began in October 2019 with a 33-month enrolment period and an estimated completion in November 2022. The approved protocol is version 7, 28 June 2021.

## Discussion

The MELRIV-1 trial is the first in human evaluation of the [^131^I]ICF01012 TRT in metastatic malignant melanomas. This study aims to determine the recommended dose of [^131^I]ICF01012 to administer for the treatment of patients with pigmented metastatic melanoma (binding [^131^I]ICF01012).

[^131^I]ICF01012 is a melanin-targeting compound allowing both imaging and TRT [18]. Even though targeting melanin to destroy melanoma cells is a seducing approach, some concerns exist and have been taken in account for this study design. Firstly, melanoma cells can lose the ability to synthesize melanin during the dedifferentiation process [30]. BZA2 phase 3 studies showed that approximately 50% of metastatic lesions did not capture melanin radiotracer [17]. Secondly, melanin is also present in healthy organs and tissues, as skin (keratinocytes, nævi), eyes (retinal pigment epithelium), brain (basal ganglia), heart, and adipose tissues (morbid obesity) [31]. Moreover, classically, radiation sensitive organs in TRT are red bone marrow, kidneys and liver. A radiosensitivity inter-patient variation of 20% dose can exist [32].

The design chosen in this trial was based on a theranostic approach with a pre-treatment dosimetry, with an infra-therapeutic dose of the [^131^I]ICF01012 that permits to select the patients who may benefit of a therapeutic dose of [^131^I]ICF01012, with at least one tumour lesion with [^131^I]ICF01012 uptake and an acceptable AD to healthy organ. Radiotoxicity must be minimized with a pre-treatment dosimetry. A precise dosimetry, i.e. the quantification of energy deposed in tumour and non-target organs, is the main concern for TRT evaluation in a clinical trial [21, 23].

The used of radiolabelled benzamides may constitute a therapeutic solution for melanomas resistant to therapy (for review see [10]). Compared to external radiation beam therapy, TRT allows a systemic treatment of a polymetastatic disease. [^131^I]ICF01012 is an original strategy for targeting pigmented lesions, but also unpigmented metastases through a potential abscopal effect [25].

## Data Availability

Data sharing is not applicable to this article as no datasets were yet generated or analysed during this ongoing study.

## Abbreviations

AD: Absorbed dose
ALP: Alkaline phosphatase
ALT: Alanine amino transferase
ANSM: Agency for the Safety of Medicines and Health Products
AST: Aspartate amino transferase
CRM: Continual Reassessment Method
CTCAE: Common Terminology Criteria for Adverse Events
DLT: Dose-limiting toxicity
ECG: Electrocardiogram
fT3: Free triiodothyronine
fT4: Free thyoroxine
GGT: Gamma-glutamyltransferase
IDMC: Independent Data Monitoring Committee
LDH: Troponin Lactate dehydrogenase
LVEF: Left ventricular ejection fraction
MIRD: Medical Internal Radiation Dose Committee
OCT: Ocular optical coherence tomography
SPECT-CT: Single Photon Emission Computed Tomography/Computed Tomography
TRT: Targeted radionuclide therapy
TSH: Thyroid-stimulating hormone
WBDC: Web-Based Data Capture
